# Comparative Evaluation of Nalbuphine and Fentanyl for Attenuation of Pressor Response to Laryngoscopy and Tracheal Intubation in Laparoscopic Cholecystectomy

**DOI:** 10.7759/cureus.15142

**Published:** 2021-05-20

**Authors:** Mohamed Kassim Akheela, Alka Chandra

**Affiliations:** 1 Department of Anaesthesiology and Critical Care, North Delhi Municipal Corporation Medical College and Hindu Rao Hospital, New Delhi, IND

**Keywords:** fentanyl, pressor response, nalbuphine

## Abstract

Background

In this study, our primary aim was to compare the efficacy of fentanyl and nalbuphine in attenuating the pressor response to laryngoscopy and tracheal intubation in patients undergoing laparoscopic cholecystectomy under general anesthesia. The secondary aim was to observe hemodynamic response to pneumoperitoneum and to study the level of sedation using the Richmond Agitation-Sedation Scale (RASS).

Methodology

A total of 180 patients belonging to the American Society of Anesthesiologist Physical Status class I/II scheduled to undergo elective laparoscopic cholecystectomy under general anesthesia were divided into two groups of 90 each. group A received intravenous nalbuphine 0.2 mg/kg and group B received intravenous fentanyl 2 μg/kg, five minutes before induction of anesthesia. Technique of anesthesia was standardized for all patients in the study. Heart rate (HR), systolic blood pressure (SBP), diastolic blood pressure (DBP), and mean arterial pressure (MAP) were recorded before giving the study drug; before induction; immediately after intubation; at one, three, and five minutes after intubation; before creating pneumoperitoneum; 15 minutes after creating pneumoperitoneum; and five minutes after release of pneumoperitoneum. Preoperative and postoperative sedation scoring was done using RASS.

Results

Immediately after intubation, HR was significantly higher in group A (p = 0.016). Both groups showed a rise in SBP immediately after intubation. Group A showed a significantly higher SBP in comparison to group B (135.97 ± 13.02 vs. 130.04 ± 13.33; p = 0.003). The DBP and MAP showed a similar trend. At one, three, and five minutes after intubation, HR, SBP, DBP, and MAP were similar between the groups. Post-extubation sedation score was significantly higher in group A (p < 0.0001).

Conclusions

We found that fentanyl was more effective than nalbuphine in attenuating the pressor response to laryngoscopy and tracheal intubation in patients undergoing laparoscopic cholecystectomy under general anesthesia. There was no significant difference observed between nalbuphine and fentanyl in the hemodynamic response to pneumoperitoneum. The depth of sedation post-extubation was significantly greater with nalbuphine.

## Introduction

Laryngoscopy, endotracheal intubation, and other airway manipulations are noxious stimuli that may induce profound changes in cardiovascular physiology, primarily through reflex responses [1]. The rise in pulse rate and blood pressure are usually transitory, variable, unpredictable, and can have detrimental consequences such as myocardial ischemia and cerebral hemorrhage. Laparoscopic surgeries form a crucial part of today’s surgical practice but pose a challenge due to significant hemodynamic alterations contributing to elevated heart rate (HR), mean arterial pressure (MAP), and increased systemic and pulmonary vascular resistance along with reduced cardiac output. Such hemodynamic changes predispose the myocardium in vulnerable patients to ischemic changes. Many techniques have been evaluated to attenuate the adverse hemodynamic response such as increasing the depth of anesthesia using drugs such as opioid analgesics, α-2 adrenoreceptors agonists, intravenous lidocaine, beta-blockers, vasodilators, and calcium channel blockers [2]. These agents have been used with varying results and are associated with their own inherent side effects. There is no single drug with minimal side effects which is cost-effective and available without a license. Hence, anesthesiologists are in constant search for the safest and the most efficient drug which can prevent the exaggerated hemodynamic response to laryngoscopy and intubation.

Fentanyl is a potent μ receptor agonist with rapid onset and relatively short duration of action, minimal respiratory depression, and has the ability to provide cardiovascular stability [3]. Despite these beneficial effects, fentanyl is known to cause bradycardia, nausea, vomiting, pruritus, and muscle rigidity. Moreover, availability of fentanyl in small hospitals is restricted due to tough narcotic laws [4]. Nalbuphine is a mixed agonist/antagonist opioid being agonist at κ receptor and antagonist at μ receptor. It is reported to have a ceiling effect on respiratory depression, cardiovascular stability, longer duration of analgesia, and decreased incidence of nausea and vomiting, which makes it an ideal analgesic during anesthesia [5]. Our primary aim was to compare the effect of fentanyl and nalbuphine in attenuating the pressor response to laryngoscopy and tracheal intubation in patients undergoing laparoscopic cholecystectomy under general anesthesia. The secondary aim was to observe hemodynamic response to pneumoperitoneum, and to study the level of sedation using the Richmond Agitation-Sedation Scale (RASS).

## Materials and methods

This prospective randomized (computer-generated) study was conducted after getting approval from the institutional ethics committee. Written informed consent was obtained from the patients after providing them with the patient information sheet. The study was conducted on 180 patients of either sex belonging to American Society of Anesthesiologist Physical Status (ASA PS) class I/II scheduled to undergo elective laparoscopic cholecystectomy under general anesthesia. The patients were divided into two groups A (nalbuphine) and B (fentanyl) of 90 patients each. The minimum required sample size with 80% power of study and 5% level of significance was 86 patients in each study group. To reduce the margin of error, total sample size was 180 (90 patients per group).

A detailed pre-anesthetic check-up was done and the anesthetic procedure was explained to the patient. All patients were kept fasting overnight. Tablet alprazolam 0.5 mg was administered orally the night before surgery and intravenous ranitidine 50 mg was administered 15 minutes before surgery. The same standardized anesthesia technique was followed in all patients. After shifting the patient to the operating room, standard monitors were attached (electrocardiogram, non-invasive blood pressure, and pulse oximetry) and baseline vital parameters were recorded. Intravenous access with 18G/20G cannula was secured and a crystalloid intravenous (IV) infusion was started. All patients were administered IV midazolam 1 mg; group A received IV nalbuphine 0.2 mg/kg and group B received IV fentanyl 2 μg/kg five minutes before induction of anesthesia. Vital parameters were noted and patients’ sedation level was assessed using RASS (Table 1) at the end of five minutes.

**Table 1:** The Richmond Agitation–Sedation Scale.

Score	term	Description
+4	Combative	Overtly combative, violent, immediate danger to staff
+3	Very agitated	Pulls or removes tube(s) or catheter(s); aggressive
+2	Agitated	Frequent non-purposeful movement, fights ventilator
+1	Restless	Anxious but movements not aggressive vigorous
0	Alert and calm	
-1	Drowsy	Not fully alert, but has sustained awakening (eye-opening/eye contact) to voice (>10 seconds)
-2	Light sedation	Briefly awakens with eye contact to voice (<10 seconds)
-3	Moderate sedation	Movement or eye opening to voice (but no eye contact)
-4	Deep sedation	No response to voice, but movement or eye opening to physical stimulation
-5	Unarousable	No response to voice or physical stimulation

After pre-oxygenation for three minutes with 100% oxygen, anesthesia was induced with intravenous propofol 1-2.5 mg/kg titrated to the loss of verbal response, and intravenous vecuronium was administered at a dose of 0.1 mg/kg, following which patient was mask ventilated for three minutes. The patient was intubated by a skilled anesthetist with an appropriately sized cuffed, orotracheal tube under direct laryngoscopy. If the patient required more than one attempt for intubation/prolonged laryngoscopy (>15 seconds), he/she was excluded from the study.

Anesthesia was maintained with isoflurane and nitrous oxide 60% in oxygen and vecuronium 0.02 mg/kg as required. Positive pressure ventilation was given with a tidal volume of 6 to 8 mL/kg body weight and respiratory rate was adjusted to maintain target end-tidal carbon dioxide between 30 and 40 mmHg. After tracheal intubation, a nasogastric tube was placed to promote baseline gastric emptying of the stomach. Patients received fluid intraoperatively as per requirement. At the end of the surgery, ondansetron 4 mg was administered intravenously.

After completion of surgery, the nasogastric tube was removed and neuromuscular block was antagonized with Inj. neostigmine 0.05 mg/kg and Inj. glycopyrrolate 0.01 mg/kg intravenously. Patient was extubated after proper oropharyngeal suctioning and return of airway reflex with adequate return of muscle power. Patient was then transferred to the post-anesthesia care unit. Systolic blood pressure (SBP), diastolic blood pressure (DBP), MAP, HR and oxygen saturation (SpO_2_) were recorded before giving the study drug (baseline recording) (T1), before induction (five minutes after administration of the study drug) (T2), immediately after intubation (T3), one minute after intubation (T4), three minutes after intubation (T5), five minutes after intubation (T6), before creating pneumoperitoneum (T7), 15 minutes after creating pneumoperitoneum (T8), and five minutes after release of pneumoperitoneum (T9). Sedation level was assessed 30 minutes post-extubation using RASS. Patients were observed for side effects such as dizziness, nausea, vomiting, respiratory depression, pruritus, hypotension, and bradycardia and were treated accordingly.

Statistical analysis

Categorical variables were presented in number and percentage (%) and continuous variables were presented as mean ± standard deviation (SD) and median. Normality of data was tested by Kolmogorov-Smirnov test. If the normality was rejected, then non-parametric test was used. Statistical tests were applied as follows: Quantitative variables were compared using independent t-test/Mann-Whitney test (when the datasets were not normally distributed) between the two groups. Paired t-test/Wilcoxon signed rank test was used for comparison between pre and post. Qualitative variables were correlated using Chi-square test/Fisher’s exact test. A p-value of <0.05 was considered statistically significant. The data was entered in MS Excel spreadsheet and analysis was done using Statistical Package for Social Sciences (SPSS) version 21 (IBM Corp., Armonk, NY).

## Results

The demographic characteristics and ASA PS grade were comparable between the two groups (Table 2).

**Table 2:** Demographic characteristics. BMI: body mass index; ASA PS: American Society of Anesthesiologist Physical Status; SD: standard deviation

	Group A (Nalbuphine) (n = 90)	Group B (Fentanyl) (n = 90)	P-Value
Age, mean ± SD	35.61 ± 9.79	35.79 ± 10.3	0.931
Sex female, %	91.1	92.2	0.787
BMI (kg/m^2^), mean ± SD	24.33 ± 4.02	24.68 ± 3.6	0.608
ASA PS I/II, n	83/7	86/4	0.536

**Figure 1:**
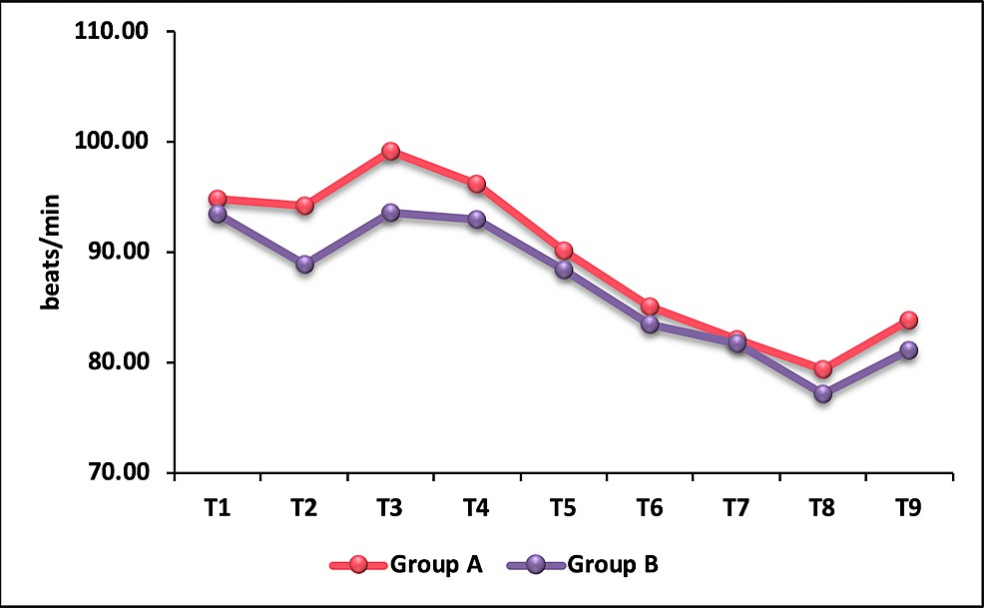
Intergroup comparison of mean heart rate.

The baseline HR was comparable between the groups. HR in the fentanyl group was significantly lower than the nalbuphine group five minutes after administration of the study drug (T2) (p = 0.015) (Table 3). Immediately after intubation (T3), HR was found to be higher in the nalbuphine group compared to the fentanyl group with a p-value of 0.016, which was statistically significant. However, HR was comparable between the groups thereafter (T4-T9) (p > 0.05) (Figure 1).

**Table 3:** Comparison of heart rate between groups A and B.

Time	Heart rate (beats/minute)		
	Group A (Nalbuphine)	Group B (Fentanyl)	P-Value
	Mean ± SD	Mean ± SD	
T1	94.83 ± 13.66	93.4 ± 15.69	0.514
T2	94.16 ± 13.98	88.88 ± 14.72	0.015
T3	99.09 ± 14.79	93.54 ± 15.71	0.016
T4	96.21 ± 14.46	92.94 ± 17.03	0.167
T5	90.13 ± 14.23	88.41 ± 15.13	0.433
T6	85.06 ± 12.43	83.42 ± 14.04	0.410
T7	82.13 ± 12.37	81.74 ± 13.82	0.843
T8	79.39 ± 12.91	77.19 ± 12.77	0.252
T9	83.82 ± 12.03	81.06 ± 13.03	0.141

**Figure 2:**
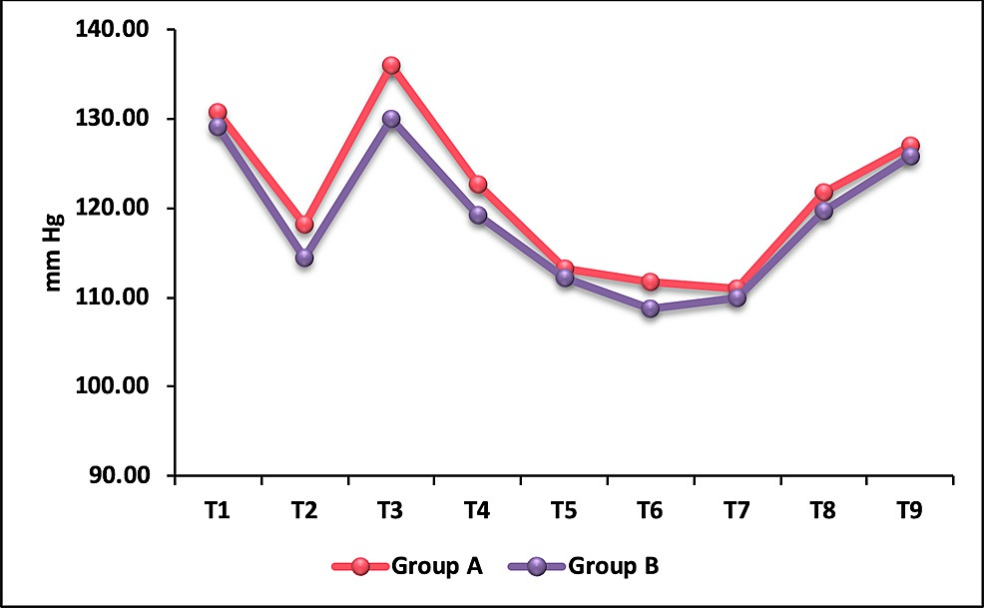
Intergroup comparison of mean systolic blood pressure.

The mean SBP was comparable between the groups before administration of the study drug. At T2, although SBP decreased in both the groups, it was significantly lower in group B compared to group A. On statistical analysis, p-value was found to be 0.004 (Table 4). Immediately after intubation (T3), both groups showed an increase in SBP, with the nalbuphine group showing a higher SBP compared to the fentanyl group (Figure 2), which was statistically significant with a p-value of 0.003. However, from T4 to T9 the mean SBP values were comparable between the groups (p > 0.05) and were not statistically significant.

**Figure 3:**
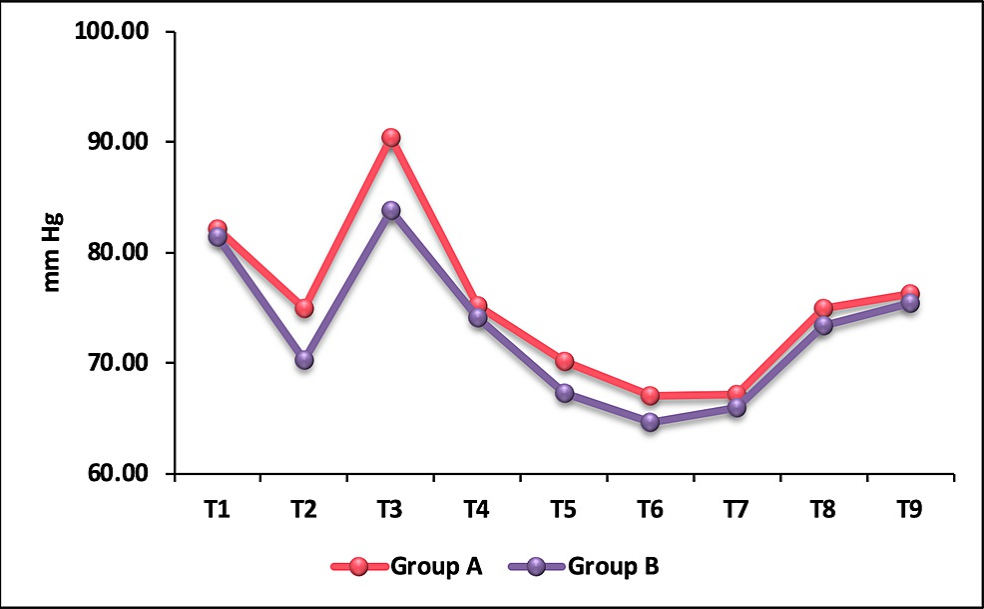
Intergroup comparison of mean diastolic blood pressure.

The baseline mean DBP was comparable between the groups (p = 0.486). At T2, that is, five minutes after administration of the study drug, DBP decreased in both the groups, but it was significantly lower in group B compared to group A with a p-value of 0.0001. Immediately after intubation (T3), both groups showed an increase in DBP, with the nalbuphine group showing a higher DBP compared to the fentanyl group, which was statistically significant with a p-value of 0.0002 (Table 4). However, thereafter there was no significant difference in DBP between the groups (p > 0.05) (Figure 3).

**Figure 4:**
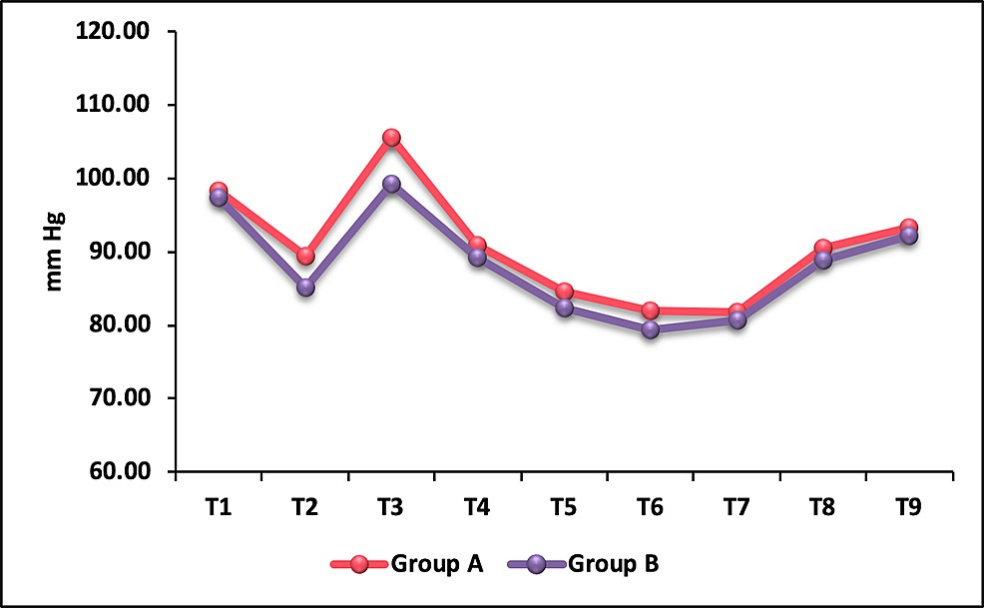
Intergroup comparison of mean arterial pressure.

The baseline MAP was comparable between the groups. There was a decrease in MAP at T2 in both the groups, but it was significantly lower in group B (fentanyl) compared to group A (nalbuphine). On statistical analysis, p-value was found to be 0.0001 (Table 4). Immediately after intubation (T3), although both groups showed an increase in MAP, the nalbuphine group had a higher MAP compared to the fentanyl group, which was statistically significant with a p-value of 0.0002. However, at one, three, and five minutes after intubation, before creating pneumoperitoneum, 15 minutes after creating pneumoperitoneum, and five minutes after release of pneumoperitoneum (T4-T9), the MAPs were comparable between the groups (p > 0.05) (Figure 4).

**Table 4:** Comparison of SBP, DBP, and MAP between groups. SBP: systolic blood pressure; DBP: diastolic blood pressure; MAP: mean arterial pressure

Time	SBP (mmHg)			DBP (mmHg)			MAP (mmHg)		
	A	B	P-Value	A	B	P-Value	A	B	P-Value
	Mean ± SD	Mean ± SD		Mean ± SD	Mean ± SD		Mean ± SD	Mean ± SD	
T1	130.69 ± 8.61	129.08 ± 9.17	0.226	82.08 ± 6.79	81.4 ± 6.23	0.486	98.28 ± 6.32	97.29 ± 5.76	0.274
T2	118.22 ± 8.03	114.48 ± 9.21	0.004	75 ± 8.51	70.3 ± 7.27	0.0001	89.41 ± 7.46	85.03 ± 6.96	0.0001
T3	135.97 ± 13.02	130.04 ± 13.33	0.003	90.44 ± 11.34	83.77 ± 12.36	0.0002	105.62 ± 11.18	99.19 ± 11.63	0.0002
T4	122.61 ± 11.68	119.26 ± 12.76	0.067	75.13 ± 10.3	74.09 ± 10.48	0.501	90.96 ± 10.08	89.18 ± 10.47	0.246
T5	113.27 ± 12.77	112.24 ± 10.54	0.559	70.19 ± 11.08	67.31 ± 9.97	0.069	84.55 ± 10.25	82.29 ± 9.35	0.124
T6	111.71 ± 11.77	108.73 ± 9.3	0.061	67.07 ± 13.15	64.61 ± 9.75	0.156	81.95 ± 11.99	79.32 ± 8.91	0.097
T7	111.01 ± 11.2	110 ± 8.87	0.503	67.2 ± 11.9	65.98 ± 11.64	0.487	81.8 ± 10.74	80.65 ± 10.09	0.459
T8	121.7 ± 8.7	119.71 ± 10.83	0.176	74.98 ± 9.49	73.36 ± 10.11	0.269	90.55 ± 8.44	88.81 ± 9.01	0.182
T9	127.01 ± 8.69	125.76 ± 7.6	0.304	76.32 ± 8.45	75.4 ± 7.85	0.449	93.22 ± 8	92.18 ± 7.07	0.36

After administration of the study drug, RASS score was -2, -1, and 0 in 18.9%, 21.1%, and 60% patients in group A and 20%, 18.9%, and 61.1% patients in group B, respectively (Table 5). This was not statistically significant. After extubation, 54.4% of patients in the nalbuphine group had a RASS score of -2 whereas none of the patients in the fentanyl group had a score of -2. RASS score was 0 in 13.3% and 86.7% of patients in group A and group B, respectively. This difference between the groups was statistically significant with p-value of <0.0001 (Table 6).

**Table 5:** Comparison of RASS score before induction. RASS: Richmond Agitation–Sedation Scale

RASS score	Group A (Nalbuphine)	Group B (Fentanyl)	P-Value
	n (%)	n (%)	
-2	17 (18.89%)	18 (20.00%)	0.928
-1	19 (21.11%)	17 (18.89%)	
0	54 (60.00%)	55 (61.11%)	
Total	90 (100%)	90 (100%)	

**Table 6:** Comparison of RASS score after extubation. RASS: Richmond Agitation–Sedation Scale

RASS score	Group A (Nalbuphine)	Group B (Fentanyl)	P-Value
	n (%)	n (%)	
-2	49 (54.44%)	0 (0.00%)	<0.0001
-1	29 (32.22%)	12 (13.33%)	
0	12 (13.33%)	78 (86.67%)	
Total	90 (100%)	90 (100%)	

There was no statistically significant difference in adverse effects between the groups. Intraoperative bradycardia (HR less than 60 beats/minute) was seen in five patients of group A and six patients of group B. Three patients in the nalbuphine group and two in the fentanyl group developed nausea and vomiting postoperatively.

## Discussion

Tracheal intubation is still the gold standard of airway management in resource-limited hospitals for general anesthesia. Intubation preceded by laryngoscopy are intense nociceptive stimuli that induce sympathetic response which is responsible for tachycardia, hypertension, and arrhythmias [6]. In the absence of any measures to prevent the hemodynamic response, the HR and BP can increase much above acceptable limits. These changes are maximum one minute after intubation and last for around five to ten minutes [7].

Fentanyl attenuates the cardiovascular response by its action on opioid receptors and by decreasing sympathetic outflow [8]. Optimal time of administration of fentanyl is five minutes before laryngoscopy and intubation, as described by Ko et al. [9]. Mixed opioid agonist/antagonists have also been studied and their efficacy on prevention of hemodynamic response to airway manipulation is still debatable. The desirability of agents with partial antagonist activity lies in the possibility of a decrease in abuse potential and a limitation to the extent of side effects, particularly respiratory depression. The analgesic potency of nalbuphine equals that of morphine on a milligram basis. The greatest advantage with nalbuphine is its ceiling effect on respiratory depression when compared to pure opioid agonists. Nath et al. [10] compared two doses of nalbuphine (group 1: 0.1 mg/kg and group 2: 0.2 mg/kg) and found better hemodynamic control with higher dose of nalbuphine. Chawda et al. [11] showed that nalbuphine prevented hemodynamic response associated with intubation when administered at a dose 0.2 mg/kg five minutes before laryngoscopy.

Although these drugs have been individually studied, there is paucity of literature comparing the effects of fentanyl and nalbuphine on pressor response to laryngoscopy, intubation, and pneumoperitoneum. The present study compared the efficacy of IV nalbuphine 0.2 mg/kg and fentanyl 2 μg/kg administered five minutes prior to induction of anesthesia for attenuating the hemodynamic response associated with laryngoscopy, tracheal intubation, and pneumoperitoneum.

The baseline HR was comparable between the groups. HR in fentanyl group was significantly lower than nalbuphine group at (T2) with a p-value of 0.015. Bhandari et al. [12] conducted a similar study and observed a decrease in mean HR after drug administration in the fentanyl group as opposed to increase in HR in the nalbuphine group. Sharma and Parikh [13] compared nalbuphine and fentanyl and found that both drugs showed almost an equal rise in HR at intubation. In both the groups, HR remained above baseline for up to 5-10 minutes after intubation, which could have been due to the effect of premedication with glycopyrrolate. Indira et al. [14] observed a significant increase in HR in nalbuphine group at one minute and three minutes post intubation, while it was comparable between the groups thereafter.

In our study, there was no appreciable change in HR from baseline five minutes after administration of nalbuphine but a significant fall in HR from baseline was noted in the fentanyl group. A significantly higher HR post intubation (T3) was observed in nalbuphine group in comparison to fentanyl group where it was closer to baseline. Though nalbuphine showed an accentuated response to laryngoscopy and intubation compared to fentanyl, the rise was transient and return to baseline in both the groups was noted at one-minute post intubation. There was no significant difference between the groups at and after one-minute post intubation.

The mean SBP, DBP, and MAP before administration of study drugs were comparable between the groups. SBP was significantly lower in the fentanyl group compared to the nalbuphine group five minutes after administration of the study drug. Immediately after intubation (T3) a significantly higher SBP was seen with nalbuphine compared to fentanyl with a p-value of 0.003. A similar result was observed by Sharma and Parikh [13]. They observed higher SBP in the nalbuphine group than the fentanyl group at one, three, five, 10- and 15-minutes post intubation, whereas in our study at one-minute post intubation and after that SBP was below baseline and comparable between the groups with a p-value of >0.05. Buchh et al. [15] compared nalbuphine and fentanyl and observed a non-significant fall in SBP in both the groups following administration of test drugs. They found a rise in SBP immediately after intubation which was the highest in the fentanyl group and did not return to baseline throughout the study period, whereas in the nalbuphine group it remained higher than baseline only until five minutes after intubation. In our study, the highest SBP was noted in the nalbuphine group immediately after intubation, whereas in the fentanyl group, it was comparable to the baseline during intubation. Thereafter throughout the study period (T4-T9), the SBP was significantly lower than the baseline in both the groups.

The basal DBP was comparable between the groups. Immediately after intubation, the DBP was significantly higher in the nalbuphine group compared to the fentanyl group. The nalbuphine group showed an increase of 10.78% from baseline compared to 3.38% of fentanyl. During post-intubation study period, DBP was significantly below baseline in both the groups. The MAP in both groups followed a trend similar to that of SBP and DBP. After administration of study drugs, a decrease in MAP in both groups was observed. Following intubation, a significant transient rise in MAP was observed in the nalbuphine group compared to the fentanyl group, which dropped to below baseline value at one-minute post intubation and remained lower than baseline thereafter throughout the study period. Indira et al. [14] reported a brief rise in MAP in the nalbuphine group one-minute post intubation, following which non-significant difference in MAP between the groups was reported.

Buchh et al. [15] observed highest MAP immediately after intubation in the fentanyl group which was statistically significant. However, at one-minute post intubation until 15 minutes, MAP was comparable between the groups. In our study, the highest MAP was recorded in the nalbuphine group immediately after intubation, and during post-intubation study period, there was no significant difference in MAP between the groups. Khanday et al. [16], in his study comparing fentanyl and nalbuphine for attenuation of hemodynamic response, found a fall in SBP, DBP, and MAP from baseline in the nalbuphine group before intubation. After endotracheal intubation, significant elevation was observed in both the groups, with higher values being noted in the nalbuphine group. Similar findings were observed in our study. The initial fall in all hemodynamic parameters in the nalbuphine group could have been because of its strong kappa agonistic action. Rise in hemodynamic parameters following intubation was due to sympathoadrenal stimulation.

A study by Madhu et al. [17] comparing nalbuphine and fentanyl in patients undergoing laparoscopic appendicectomy found no significant difference in HR, SBP, DBP, or MAP between the groups after creation of pneumoperitoneum. Similarly, in our study, hemodynamic parameters were comparable between the two groups after creation of pneumoperitoneum.

RASS scores after administration of study drugs were comparable between the groups. However, at 30 minutes after extubation, 54.4% of patients in the nalbuphine group had a RASS score of -2 compared to none in the fentanyl group, reflecting the sedative effect of nalbuphine due to kappa agonistic action [18]. Madhu et al. [17] reported significantly higher sedation scores in the nalbuphine group compared to fentanyl group after extubation in post-anesthesia care unit. However, they had assessed sedation using Pasero Opioid-Induced Sedation scale.

Intraoperative bradycardia was observed in five patients in the nalbuphine group and six patients in the fentanyl group which was not statistically significant. This could be attributed to peritoneal stretching owing to pneumoperitoneum [19]. Postoperative nausea and vomiting was reported in three patients in the nalbuphine group and two patients in the fentanyl group. None of the patients in our study developed pruritus or respiratory depression. The limitations of our study were that we only studied ASA PS I/II patients, stress mediators such as endogenous plasma catecholamines were not measured, invasive arterial blood pressure monitoring was not done, and the study was limited to patients undergoing laparoscopic cholecystectomy.

## Conclusions

In our study, we found that fentanyl was more effective than nalbuphine in attenuating the pressor response to laryngoscopy and tracheal intubation in patients undergoing laparoscopic cholecystectomy under general anesthesia. There was no significant difference observed between nalbuphine and fentanyl in the hemodynamic response to pneumoperitoneum. The depth of sedation post extubation was significantly greater with nalbuphine.
